# Supplementary material for: Typical pneumonia among human immunodeficiency virus-infected patients in public hospitals in southern Ethiopia
Source: PLoS One. 2024 Jul 30;19(7):e0307780. doi: 10.1371/journal.pone.0307780 (PMC11288459; doi:10.1371/journal.pone.0307780)
Supplement: Attachment — Submitted filename: Review report-Plos One.pdf [file pone.0307780.s002.pdf]

Review report (PLOS ONE)

Date: 21 January, 2024

The manuscript entitled '**Typical Pneumonia among HIV infected patients in public Hospitals, Southern Ethiopia**' by *Aklilu et al* attempts to inform the prevalence of drug resistant bacteria causing pneumonia among HIV infected subjects in the given study area. I am impressed by the ample sample size, coherence in writing and ideas, and the compelling findings. I therefore support this article to appear in the 'Plos One'. However, the authors may want to improve on the following sections unless the article is not acceptable in the present form.

Comments

Introduction

1. This section sounds good which has been able to articulate ideas. I suggest authors add a or two sentence(s) on the importance of pyogenic bacteria among HIV-related infections.
2. L20-23: The sentence is not clear. I suggest authors rewrite the sentence.

Methods

1. There seem to be some structural differences among the two types of hospitals: general and primary. How are the HIV-related cases managed differently in these hospitals? I suggest authors describe it in a or two sentence(s).
2. I suggest authors provide figures of the subjects on each of the excluded criteria and total approached subjects.
3. Include methods (phenotypic and confirmatory) describing ESBL and carbapenem resistant tests.

Discussion

1. I suggest authors add a small paragraph on the strengths, limitations, and clinical as well as policy implications of the study.

Conclusion

1. This section needs a major revision. I suggest authors trim the description and rewrite the more meaningful conclusion.

L 434: Citation format needs to be adjusted.

Table 4: Write the full form of all abbreviations.

Above all, the article needs further improvement in standard English and grammatical errors.
