# Supplementary material for: Typical pneumonia among human immunodeficiency virus-infected patients in public hospitals in southern Ethiopia
Source: PLoS One. 2024 Jul 30;19(7):e0307780. doi: 10.1371/journal.pone.0307780 (PMC11288459; doi:10.1371/journal.pone.0307780)
Supplement: Attachment — Submitted filename: Responses to the Reviewers.pdf [file pone.0307780.s005.pdf]

Responses to the Reviewers' comments	
We carefully considered the reviewer's comments and have revised the article. We are very much thankful to the Reviewers whose suggestions substantially improved the quality of the manuscript we believe.	
Editorial Corrections:	**Response**
Journal Requirements:<br><br>Please review your reference list to ensure that it is complete and correct. If you have cited papers that have been retracted, please include the rationale for doing so in the manuscript text or remove these references and replace them with relevant current references. Any changes to the reference list should be mentioned in the rebuttal letter that accompanies your revised manuscript. If you need to cite a retracted article, indicate the article's retracted status in the References list and also include a citation and full reference for the retraction notice.	**Reference No. 14 is removed and replaced with an appropriate one in the revised manuscript.**<br><br>**Older reference- Retracted is removed**<br><br>14. Hartung TK, Chimbayo D, van Oosterhout JJ, Chikaonda T, van Doornum GJ, Claas EC, Melchers WJ, Molyneux ME, Zijlstra EE. Etiology of suspected pneumonia in adults admitted to a high-dependency unit in Blantyre, Malawi. Am J Trop Med Hyg. 2011 Jul;85(1):105-12. Doi: 10.4269/ajtmh.2011.10-0640.<br><br>**Replaced with New reference**<br><br>14. Olaru ID, Tacconelli E, Yeung S, Ferrand RA, Stabler RA, Hopkins H, Aiken AM, Kranzer K. The association between antimicrobial resistance and HIV infection: a systematic review and meta-analysis. Clin Microbiol Infect. 2021 Jun;27(6):846-853. doi: 10.1016/j.cmi.2021.03.026.
Reviewers comment- Reviewer 2	**Responses to the Reviewers' comments**
I appreciate your feedback on my suggestions. But you should consider my previous comment: "Blood agar plates should be incubated in a 5-10% carbon dioxide generating candle jar at 37˚C for 24 hours like a chocolate agar plate.	Thanks for your encouraging comments.<br><br>Corrected in the revised manuscript as '*The blood and chocolate agar plates were incubated (5-10% CO_2) in a candle jar at 37° C for 24-48 hours.*'
Again, in your manuscript, you have used the word "pyogenic" repeatedly. I'm not satisfied with the way you mentioned the bacterial species that cause productive coughs. I think you should differentiate pyogenic bacteria, which means pus-forming bacteria such as S. aureus and S. pyogenes. Sputum is expectorated from the lungs and composed of mucus that may contain pus (dead WBCs), blood, fibrin, or	We have considered your comment carefully.<br><br>The word 'pyogenic' is entirely removed from the revised manuscript as per your suggestion.

bacteria. I advised you to remove this word. Thank you!	
Reviewer 4	
Abstract<br>-Methods section: What type of culture media and biochemical tests are used for bacterial identification?<br>-Conclusion section: what do you recommend?	Thanks.<br><br>We have used selective and enriched media for the cultivation of bacteria. They are 5% sheep blood agar, chocolate agar, mannitol salt agar, and MacConkey agar (Oxoid, Basingstoke, Hampshire, UK).<br><br>Details of Biochemical Testes are mentioned in the materials and method section under the subtitle **Bacterial isolation.**<br>**Due to the restrictions in word count, we are unable to mention the complete details in the abstract.**<br><br>The recommendation is included in the conclusion section of the abstract in the revised manuscript.
Materials and Methods<br>-Do you considered local availability of antibiotics for AST while you choose antibiotics in addition to following CLSI guidelines?	Thanks for your comment.<br><br>Yes, we have selected antibiotics based on **CLSI guidelines** and also by considering the **prescription policy followed in our study settings**, which is exactly the same as the national policy.<br><br>This is indicated in the methodology section under **Antimicrobial Susceptibility Tests as** per your comments.
Discussion<br><br>-What factors attribute for shift in prevalence from gram positive to gram negative bacteria?<br><br><br><br><br><br><br><br><br><br><br>-Previously S. aureus is most prevalent in typical pneumonia, in your study K. pnuemoniae was most prevalent, So how	Thanks for your queries<br><br>In our study, members of the *Enterobacteriaceae* family were the predominant aerobic bacteria found. The lack of awareness of healthcare workers, as well as patients, on droplet precautions to be taken and the high environmental burden of Gram-negative bacteria, could be the factors attributed to the paradigm shift from Gram positives to Gram negatives.<br>We haven't separately elucidated the aetiology of hospital/community-acquired pneumonia and hence cannot arrive at a strong conclusion at this juncture. Further in-depth studies involving different settings and larger size study populations are required to arrive at a firm conclusion regarding the paradigm shift at a national level.

do you explain this?	Thanks for your query<br><br>Klebsiella is a typical colonizer of human mucosal surfaces of the upper respiratory tract, i.e., the oropharynx. It can cause destructive changes to human lungs when aspirated and also act as a supreme opportunist pathogen in patients with compromised immunity like HIV patients.<br>In developing countries of Africa, K. pneumonia accounts for approximately 15% of all cases of pneumonia.<br>Coming to our study, the most common causative agent was *K. pneumoniae* (25.7%), followed by *S. aureus* (17.8%).<br><br>We envisage that the emergence of antibiotic-resistant strains of K. pneumoniae, particularly the extended-spectrum β-lactamases (ESBLs) and carbapenemase-producing strains, have gained the upper hand and hence can successfully establish an infection. Predominance of K. pneumoniae in HIV patients with pneumonia is recently reported in a study done in Ethiopia itself [Tilahun et al., 2023; doi: 10.1177/20503121221145569.]<br><br>However, in the present study, we are unable to clearly figure out the exact reason for the predominance of Klebsiella. We hope that in the near future, we will be able to elucidate the reasons for the change in the spectrum of bacteria associated with typical pneumonia.
