# Supplementary material for: Typical pneumonia among human immunodeficiency virus-infected patients in public hospitals in southern Ethiopia
Source: PLoS One. 2024 Jul 30;19(7):e0307780. doi: 10.1371/journal.pone.0307780 (PMC11288459; doi:10.1371/journal.pone.0307780)
Supplement: Attachment — Submitted filename: Responses to the Reviewers.docx [file pone.0307780.s003.docx]

| **Responses to the Reviewers' comments**  We carefully considered the reviewer's comments and have revised the article. We are very much thankful to the Reviewers whose suggestions substantially improved the quality of the manuscript we believe. | |
| --- | --- |
| Editorial Corrections: | **Response** |
| Reviewers comment- Reviewer 1 | **Responses to the Reviewers' comments** |
| The manuscript entitled ‘Typical Pneumonia among HIV infected patients in public Hospitals, Southern Ethiopia’ by Aklilu et al attempts to inform the prevalence of drug resistant bacteria causing pneumonia among HIV infected subjects in the given study area. I am impressed by the ample sample size, coherence in writing and ideas, and the compelling findings. I therefore support this article to appear in the ‘Plos One’. However, the authors may want to improve on the following sections unless the article is not acceptable in the present form. | Thanks for your encouraging comments. |
| Introduction 1. This section sounds good which has been able to articulate ideas.  I suggest authors add a or two sentence(s) on the importance of pyogenic bacteria among HIV-related infections. | Thanks for the comment.  Thanks. We included a few lines in the revised manuscript. |
| 2. L20-23: The sentence is not clear. I suggest authors rewrite the sentence. | Corrected in the revised manuscript |
| Methods 1. There seem to be some structural differences among the two types of hospitals: general and primary. How are the HIV-related cases managed differently in these hospitals? I suggest authors describe it in a or two sentence(s). | Thanks. The details are included in the revised manuscript. |
| 2. I suggest authors provide figures of the subjects on each of the excluded criteria and total approached subjects. | Thanks for your comment.  As per the inclusion criteria, 416 patients were recruited within a span of six months. Among them, only 368 were selected based on the quality of sputum samples, i.e., Barlett’s score. |
| 3. Include methods (phenotypic and confirmatory) describing ESBL and carbapenem-resistant tests. | Detailed methodology is included in the revised manuscript. |
| Discussion 1. I suggest authors add a small paragraph on the strengths, limitations, and clinical as well as policy implications of the study. | Thanks for your comments.  We agree with your comments and have added a paragraph mentioning the strengths, limitations, and clinical and policy implications as per your comment. |
| Conclusion 1. This section needs a major revision. I suggest authors trim the description and rewrite the more meaningful conclusion. | We solely considered your comment.  The conclusion section is fully revised. |
| L 434: Citation format needs to be adjusted | The citation format is corrected, and DOI is provided. |
| Table 4: Write the full form of all abbreviations. | Abbreviations are expanded as and where required. |
| Above all, the article needs further improvement in standard English and grammatical errors. | The English section of the manuscript is corrected with the aid of an English language expert. |
| Reviewer #2: Thank you for inviting me to review the manuscript. Although the research idea is very interesting, it lacks some clarity. The authors should consider the following issues: | Thanks for your valuable comments. |
| 1. There is no clear information about the type of pneumonia (Community-acquired vs hospital-acquired). | We considered your comments. We have mentioned the type of pneumonia in the objective as well as in the methodology section of the manuscript. |
| 2. There are grammar mistakes and redundancy of words (example: the word “In fact” in lines 8, 9, and 17, “Ipso facto” in line 41, “area, setting” in line 62, “opted” in line 77….etc). | Thanks, we have corrected all the grammar mistakes as mentioned by you. |
| 3. Abstract: the sentence “Sputum were collected and inspected for pyogenic bacteria following standard…..”. Why you mention pyogenic here? In my point of view, it is misunderstanding. | Thanks, we have removed it from the abstract section. |
| 4. Line 99: “Expectorated specimens (3-5ml) were collected in a sterile sputum cup (falcon tube)”.  How you collect sputum in HIV patients that are unable to produce and give sputum specimen?  Have you used sputum inducer? | Patients that are unable to produce and provide enough sputum were excluded from the study. We have already included it in the exclusion criteria.  No, we haven’t used sputum inducer. |
| Again, sputum cup and falcon tube are different materials. | Thanks, we have corrected it as a sputum cup. |
| 5. Line 104: Procedure for bacterial identification should be written in detail. | Thanks. We have provided a detailed methodology for the identification of bacteria. |
| 6. Line 107-108: Blood agar plate should be incubated in 5% carbon dioxide generating candle jar at 37˚C for 24 hours | Thanks, we have corrected it. |
| 7. Line 214: Define MDR | Thanks, we have defined the MDR. |
| Reviewer #3: This work is very important and interesting in trying to assess prevalence of typical pneumonia among vulnerable population. However, there are major revisions required to the writing style of the manuscript, especially abstract and methods. I have suggestion on the following issue. | Thanks for your valuable comments.  We considered your comments and revised the manuscript. |
| - Abstract part it says “A pretested semi-structured questionnaire was employed to collect the demographic” what does it mean semi-structured, please correct as structured. | Corrected as ‘structured questionnaire’ in the revised manuscript |
| - It says, “A total of 152 pyogenic bacteria were identified, comprising 73% (n=111) Gram negative”. Gram negatives what? Please correct Gram negative as Gram negative isolate or Gram-negative bacteria. | It was corrected as ‘Gram-negative bacteria’ as and where required. |
| - It says “Two-score (11/27) of S. aureus was MRSA, and 26.6 (n=29) and 21.1% (n=23) of Gram-negative isolates were ESBL and carbapenemase producers, respectively”. What is Two-score? | Corrected in the revised manuscript by removing ‘Two scores’. |
| And please correct the percentage of ESBL like this 26.6%. | Corrected in the revised manuscript as 26.6% |
| - All abbreviations, such as MRSA should be defined on first use, generally abbreviations is not recommended in abstract part. | Abbreviations are expanded in the revised manuscript. |
| -please correct Keywords as follows: typical pneumonia, bacterial isolates, pyogenic, antimicrobial susceptibility pattern, HIV, Arba Minch, Ethiopia. | Keywords are corrected as per your comment. |
| Introduction part -line 8-9: the author uses two times in fact, it indicated that there is self-plagiarism, so, please correct it. | It was corrected in the revised manuscript. |
| -line 10 and 21: AIDS and WHO should be used long form with abbreviations | Abbreviations are expanded in the revised manuscript. |
| Methods and Materials - There are like AMGH and DFPH please use long form. | It was corrected in the revised manuscript by giving the full form. |
| -for Bacterial isolation method are like conventional biochemical method and molecular methods are used, so which method was used for identification of bacterial species like K. pneumoniae, S. aureus E. coli, S. pyogenes, P. aeruginosa, E. aerogenes, and S. pneumoniae. Please describe all method you have used for identification for all bacterial isolates. | Thanks. A detailed methodology for the identification of bacteria is provided in the revised manuscript. |
| -How do you identify MRSA | The methodology for MRSA identification is given in the revised manuscript with appropriate reference. |
| -the authors use sputum sample but there is not mycobacterium tuberculosis in the result part, because this bacterium one of the causative agents of pneumonia, if so, please say something in the method part or inclusion and exclusion criteria. | Thanks. We have excluded those patients with TB and mentioned them in the exclusion criteria. |
| - For culture media the author must write manufactured company, and the country | Details of the manufacturing company and address were mentioned in the revised manuscript. |
| - The authors should be mentioned all antibiotics with their concentration, manufactured company, and the country | Thanks. All the antibiotics used in the study are mentioned with proper details in the revised manuscript. |
| --line 135: ATCC should be used long form with abbreviations, then you can use abbreviations | The full form of the ATCC is mentioned. |
| - Ethical considerations. What was the fate of those patients positive for pyogenic bacteria? | Thanks, Culture-positive results with antimicrobial susceptibility tests were reported promptly to the ART physicians.  There was no causality observed during the study period. |
| Result part - “Ok” but please increase the resolution of figures | We have increased the resolution. |
| -The discussion is OK. But it needs some improvements. | Thanks. We have modified the discussion section by adding a few paragraphs with updated references. |
| - Please see again the conclusions; it is not based | Thanks. We have fully revised the conclusion part. |
